# Acid shock triggers virulence surge accompanied by bacterial elongation in *streptococcus mutans*

**DOI:** 10.1038/s41598-026-52966-x

**Published:** 2026-05-12

**Authors:** Haiyun Zhou, Qingyi Shao, Jiaji Ye, Danlei Chen, Meijin Xu, Qing Ye, Dongqing Cheng

**Affiliations:** 1 School of Medical Technology and Information Engineering, Zhejiang Chinese Medical University, Hangzhou, 310053 China; 2 Department of Laboratory Medicine, Children’s Hospital, Zhejiang University School of Medicine, Hangzhou, 310052 China

**Keywords:** *Streptococcus mutans*, Pediatric Caries, Acid shock, Virulence, Bacterial elongation, Diseases, Microbiology

## Abstract

Pediatric caries is a major global oral health burden, children are particularly susceptible due to immature oral defense mechanisms, underdeveloped immune responses, and frequent exposure to sugar-rich diets that promote cariogenic bacterial activity. *Streptococcus mutans* (*S. mutans*) is widely recognized as a primary cariogenic pathogen, responsible for key virulence factors such as acid production, acid tolerance and exopolysaccharide (EPS) synthesis. Given that *S. mutans* can adapt to acidic environments through mechanisms such as the acid tolerance response (ATR), we cultured *S. mutans* in a medium supplemented with hydrochloric acid or lactic acid at pH gradients of 6, 5 and 4 to observe how its virulence changed in response to early low pH shock. *S. mutans* was found to exhibit delayed growth, increased biofilm biomass, and up-regulated expression of virulence-associated genes, accompanied by bacterial elongation under early low pH shock. It is noteworthy that the morphological changes in bacteria have a substantial impact on biofilm formation and stability. We propose that early acid shock may trigger a virulence surge through bacterial elongation, thus conferring a stronger cariogenic capacity. This finding provides critical insights into the pathogenic mechanisms of pediatric caries and offers potential targets for developing tailored preventive strategies for children.

**Supplementary Information:**

The online version contains supplementary material available at 10.1038/s41598-026-52966-x.

## Introduction

Early childhood caries (ECC) stands out as the most prevalent chronic disease in children worldwide, with its harms far exceeding simple tooth damage^1^. Beyond triggering persistent tooth pain that disturbs children’s sleep and daily activities, untreated ECC often progresses to severe complications like pulpitis and periapical periodontitis, and in extreme cases, even maxillofacial infections that can develop into life-threatening bacteremia when pathogens invade the bloodstream^2^. For preschool children, this not only impairs chewing ability but also disrupts normal nutritional absorption, which in turn hinders physical development and cognitive growth. Additionally, the chronic pain and oral disfigurement caused by caries can lead to negative emotional changes in children, such as low self-esteem and irritability, further affecting their academic performance and social interactions with peers^3^. Dental caries is driven by acidic demineralisation of enamel and dentin, where acid-producing bacteria, dietary carbohydrates and host factors interact^4^. Chronic sugar consumption disrupts host-microbe interactions, creating a microenvironment conducive to the growth of acid-producing and acid-resistant microorganisms^5^.

The main virulence factors of *Streptococcus mutans* (*S. mutans*), a major cariogenic pathogen, include acid production, acid tolerance, and exopolysaccharide (EPS) synthesis. One of its strong acid stress responses results in better acid tolerance than other oral commensals and thus a greater survival advantage in the oral cavity^6^. By maintaining an alkaline cytoplasmic environment through the alteration of plasma membrane unsaturated fatty acids, the agmatine deiminase system, malolactic fermentation, and high production of lactic acid, the end product of glycolysis, *S. mutans* outcompetes other members of the microbial community and occupies a larger ecological niche^6,7^. Acidic conditions trigger its acid-tolerance response, up-regulating stress genes and activating acid tolerance-related metabolic pathways, such as the phosphoenolpyruvate (PEP): carbohydrate phosphotransferase system (PTS)^8^ and base metabolism pathways to preserve pH homeostasis^9–11^. Moreover, *S. mutans* offers protection against the external acidic environment by forming biofilms that slow down the penetration of acid^12^. The biofilm matrix surrounding bacteria not only confers tolerance to harsh conditions but also plays a key role in causing a broad range of chronic diseases^13^.

Although the acid adaptive response of *S. mutans* has been well studied^14,15^, less attention has been given to the effects of early, intermittent acid shocks, which more closely mimic the fluctuating pH environment in pediatric caries. Furthermore, although bacterial morphology is increasingly recognized as an adaptive strategy to environmental stress^16,17^, whether morphological changes contribute to the enhanced cariogenic potential of *S. mutans* under early acid stress has not been systematically investigated.

Therefore, the present study focuses on the virulence alterations and morphological changes of *S. mutans* in response to early acid shock. Using pH gradients based on self-generated acidification observed in preliminary experiments, we integrated transcriptomic, phenotypic, and morphological evidence to investigate whether early acid shock induces bacterial elongation and whether this morphological response, by enhancing biofilm formation and stability, is associated with increased cariogenic potential. Aiming to provide a more comprehensive understanding of the early adaptive strategies of *S. mutans* and their contribution to cariogenicity in the context of early childhood caries.

## Materials and methods

### Bacterial strain and culture conditions

*S. mutans* UA159 colonies were cultured anaerobically (5% CO_2_, 37℃) in Brain Heart Infusion (BHI, OXOID, USA) agar, then in BHI broth to 10⁷ CFU/ml. Biofilms were formed in BHI containing 1% sucrose. The initial pH of the medium was adjusted to 6, 5, and 4 (± 0.2) with 1 M hydrochloric acid (Ha6/5/4) or 1 M lactic acid (La6/5/4) to construct an early low pH shock environment to set up an experimental group, unadjusted pH 7.0 served as control. This pH setting was derived from a pre-experiment which showed that *S. mutans* biofilms with 1% sucrose naturally drop to pH 4 after 48 h (Supplementary Table 1), this self-generated minimum was therefore chosen as the realistic endpoint. Hydrochloric acid was used to generate non-specific low pH stress as a control, while lactic acid was employed to simulate the physiological organic acid environment produced by *S. mutans* metabolism during caries development.

### Fluorescence microscope observation of suspended bacteria morphology

*S. mutans* cultures (OD_600_=0.5) were incubated at 37℃ under 5% CO_2_ and harvested during the Log-phase, washed in phosphate-buffered saline (PBS), stained for 5 min with 4’,6-diamidino-2-phenylindole (DAPI, excitation 364 nm, emission 454 nm), and imaged by fluorescence microscopy. The experiment was performed in three independent biological replicates, with at least five random microscopic fields captured per replicate for morphological analysis.

### Scanning electron microscope imaging

*S. mutans* biofilms grown on slides in BHI containing 1% sucrose for 24 h (5% CO_2_, 37℃) under different pH conditions (control pH 7.0, Ha6/Ha5/Ha4, and La6/La5/La4) were fixed in 2.5% glutaraldehyde, subjected to ethanol gradient dehydration, gold-sputtered, and imaged at 10 kV by scanning electron microscopy (SEM, Hitachi, Japan). For bacterial length quantification, images were analyzed using ImageJ software. Length was measured along the long axis of clearly delineated bacterial cells within chains. Three independent biofilm samples were prepared for each condition, and six representative areas were randomly selected from each sample for observation.

### Growth curve of *S. mutans* under early low pH shock

Overnight cultures were diluted into 50 mL BHI at pH 7 (control) and adjusted to 6, 5, 4 with HCl or lactic acid, then OD_600_ was read every 1–2 h for 24 h (37℃) to track growth kinetics.

### Determination of bacterial surface hydrophobicity and bacterial aggregation

After exposing each group to their respective low pH conditions for 8 h, 2 mL of each culture was pelleted by centrifugation and washed with PBS. To measure hydrophobicity, add 20% xylene to the cell suspension, shake vigorously for 2 min, and then let it stand for 15 min to allow phase separation. Measure the OD_595_ of the aqueous phase before (A0) and after (A) extraction. The percentage of hydrophobicity is calculated as follows:

1 $${\rm HPBI \% = [(A_{0}-A)/A_{0}] \times 100\% }$$

For self-aggregation, OD_595_ was measured at hour 0 of cultivation (A0) and again after incubation at 37 °C for 2 h (A2h). The percentage of aggregation was calculated as follows:

2 $${\rm Aggregation \% =[1- (A_{2h}/A_{0})] \times 100\%}$$

Three independent replicates were performed for each condition.

### Biofilm crystal violet staining for biomass detection

Biofilms were grown in 24-well polystyrene plates (Corning, USA) for 24 h under the conditions described above. After incubation, biofilms were washed with PBS, air-dried, stained with 0.2% crystal-violet for 15 min, decolored with 33% glacial acetic acid, and measured at OD_570_. Three independent experiments with triplicate wells were performed.

### Biofilm EPS and protein content assay

Biofilms were grown under the conditions described above, then collected and extracted with 1 M NaOH (2 h) for insoluble EPS, quantified by 5% phenol and sulphuric acid (OD_625_); protein measured with BCA kit (Biyun Tian, China).

### Differential gene analysis and pathway enrichment under early low pH shock

A publicly available Gene Expression Omnibus (GEO) dataset GSE55952 (GPL15711, UA159) containing 8 samples with pH 7 vs. 8 samples with pH 5 *S. mutans* arrays was retrieved via GEOquery. After normalization and annotation, differentially expressed genes (DEGs) (pH 5 vs. 7, *P* < 0.05, |log2 FC| > 1) were identified with LIMMA package of the R software, visualized in volcano plots and functionally annotated by Gene Ontology (GO) enrichment (FDR < 0.01).

### Incorporation of the PPI network

Based on the DEGs identified above, potential interactions between their encoded proteins were retrieved via the Search Tool for the Retrieval of Interacting Genes/Proteins (STRING) (https://string-db.org/), predicting the protein-protein interaction (PPI) networks. Subsequently, molecular interaction networks were built and visualized using the Cytoscape program.

### Primer design and analysis of gene expression by real-time quantitative PCR

DEGs were validated by real-time quantitative polymerase chain reaction (RT-qPCR). Gene primer sequences are shown in Supplementary Table 2. Biofilms were cultured as described above and the RNA from biofilms was extracted by the Total RNA Isolation Kit (Vazyme, China), reverse-transcribed using HiScript II Q RT SuperMix for qPCR (Vazyme, China), and amplified using Applied Biosystems 7500 Real-Time PCR Systems and Taq Pro Universal SYBR qPCR Master Mix (Vazyme, China). Gene expression was calculated via 2^−∆∆Ct^. Three independent biological replicates were performed.

### Statistical analysis

Statistical analyses were performed using GraphPad Prism 9.0. Data are presented as mean ± SD from at least three independent experiments performed in triplicate. Normality was assessed using the Shapiro-Wilk test; non-parametric tests were applied for non-normally distributed data. For comparisons between two groups, Student’s t-test (normal distribution) or Mann-Whitney U test (non-normal distribution) was used. For multiple group comparisons, one-way ANOVA followed by Tukey’s HSD post-hoc test was employed. A p-value of less than 0.05 was considered statistically significant (*: *P* < 0.05, **: *P* < 0.01, ***: *P* < 0.001).

## Results

### The prolongation of the bacterium and the slowing down of division and proliferation of planktonic *S. mutans* under early low pH shock.

**Fig. 1:**
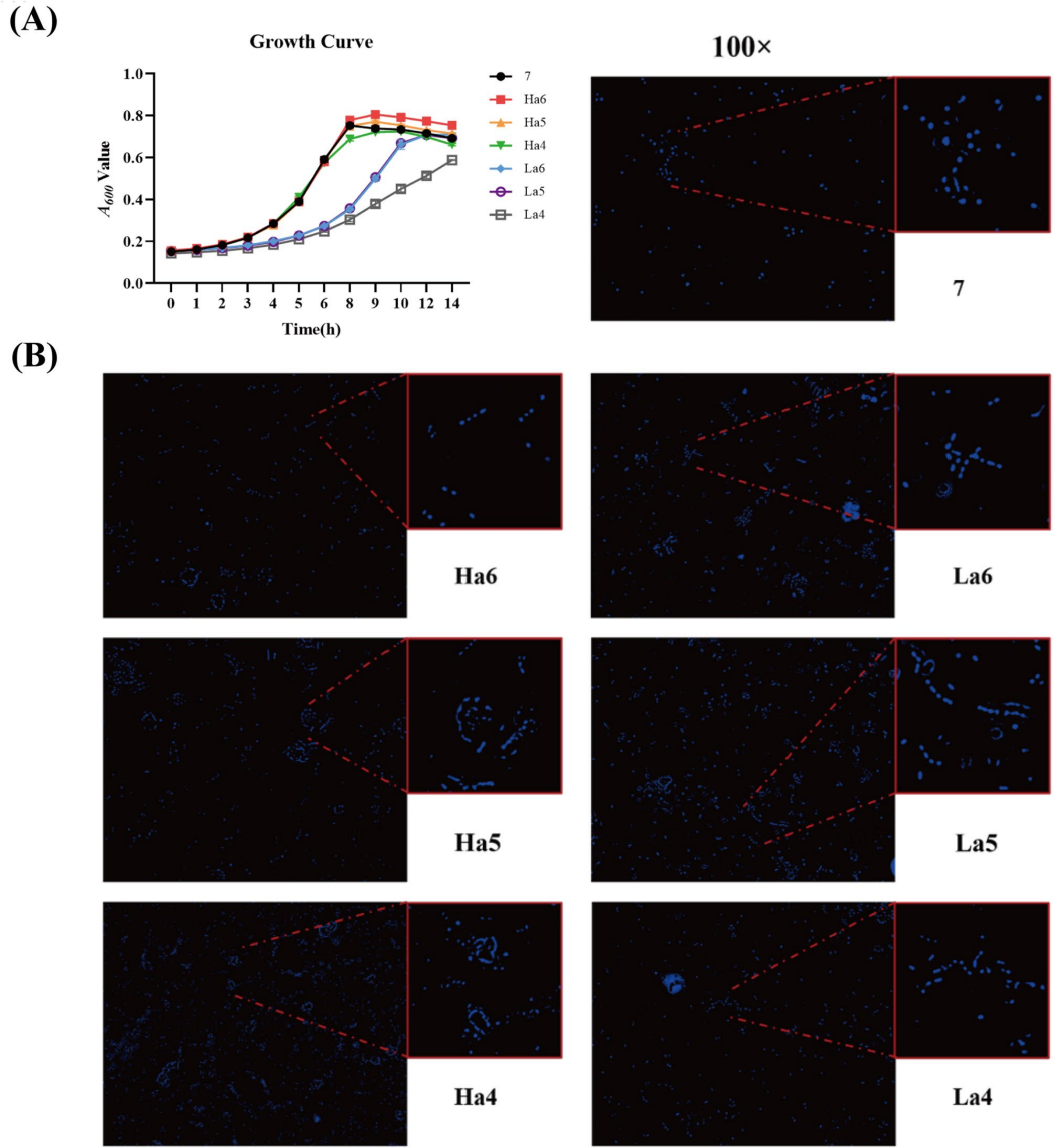
Under early low pH shock (**A**) the growth curves of *S. mutans* and (**B**) bacterial arrangement status of planktonic *S. mutans*, the red dashed line shows the proportionally enlarged part of the right red box.

To assess the impact of early acid shock on planktonic growth and morphology, we examined growth kinetics and cellular appearance. No change in the growth of *S. mutans* was observed in Ha6, Ha5 and Ha4 compared to the pH 7 group. In contrast, the growth of *S. mutans* was slowed down in La6, La5, La4, mainly in the form of delayed entry into the logarithmic growth phase with a decrease in the final bacterial concentration. Cultures grown at pH 7 entered the logarithmic growth phase at approximately 3 h, whereas those grown at lower pH due to the addition of lactic acid entered logarithmic growth at 5 h. No difference in final bacterial concentration was observed between the pH 6 and 5 groups under lactic acid compared to pH 7 group, while the final bacterial concentration was lower at pH 4 group (Fig. 1A). Observation of pre-stained suspended bacteria by fluorescence microscopy revealed that at pH 7 group, the bacteria were distributed in a punctate manner, whereas *S. mutans* showed either a prolonged or annular distribution after the pH decreased (Fig. 1B).

#### Bacterial elongation in *S. mutans* biofilms under early low pH shock

**Fig. 2:**
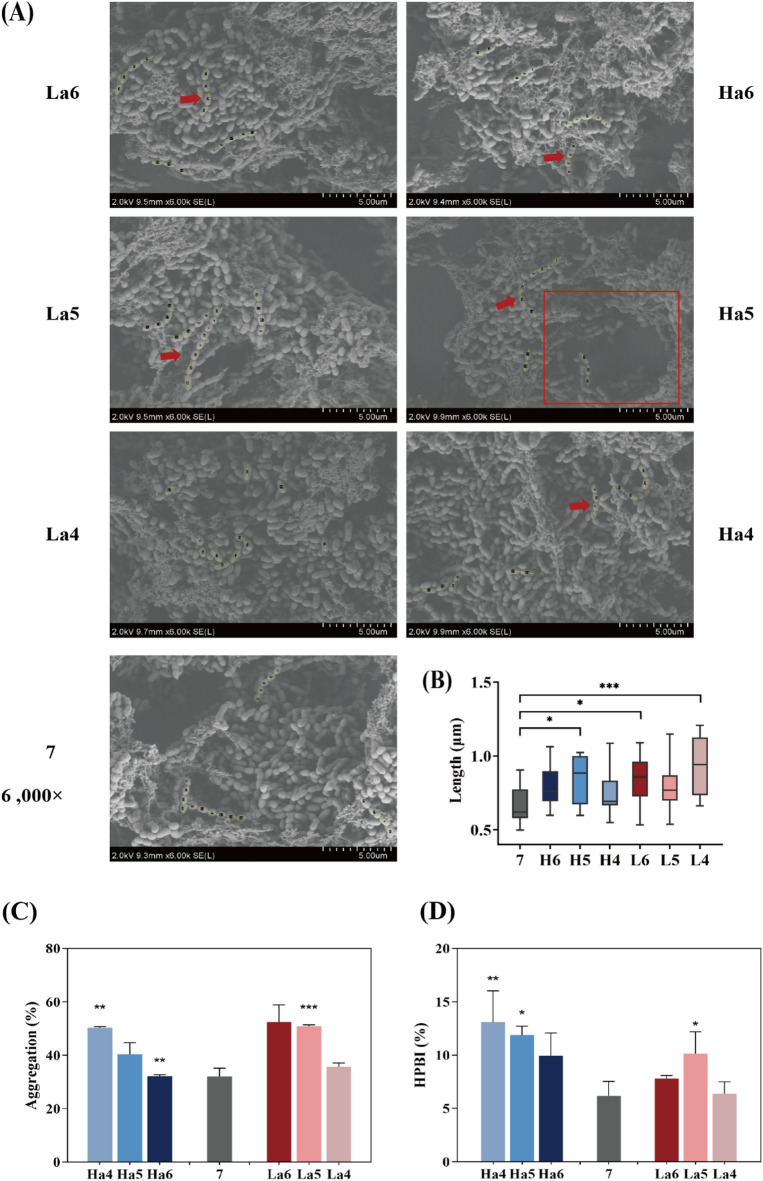
The elongation of *S. mutans* in biofilms under early low pH shock. (**A**) At 6000× magnification of electron microscope, the red arrows indicate *S. mutans* in biofilms with elongated bacterial bodies, and the red boxes indicate *S. mutans* in biofilms arranged to form a pore-like shape; the numbers delineated in yellow are randomly selected bacterial organisms that are distributed in chains; (**B**) Length of bacteria obtained from the chain-like *S. mutans* in A (yellow delineated portion); (**C**) Aggregation rates and (**D**) Hydrophobicity rates under early low pH shock planktonic *S. mutans*. Comparison with the pH 7 group: *: *P* < 0.05, **: *P* < 0.01, ***: *P* < 0.001, not labelled i.e. no significant difference seen.

To determine whether early acid shock induces morphological changes in biofilm-associated cells, we examined biofilm architecture by electron microscopy. Electron microscopy showed that at pH 7, some of the *S. mutans* in the biofilm were in short chains, mostly 4–5 organisms forming a chain (defined as “short chains”), whereas at pH 6/5/4 under hydrochloric acid (Ha6, Ha5, Ha4), the *S. mutans* organisms were prolonged in the biofilm and the biofilm architecture exhibited a pore-like shape (red box in Fig. 2A). Meanwhile, *S. mutans* in the biofilms of the La6 and La5 groups was also characterized by a prolonged aspect ratio of the bacterium and the formation of longer chain-like structures exceeding eight cells (defined as “long chains”), but this was not observed in the La4 group, where only a uniform bacterial morphology was observed. Further length measurements were performed using ImageJ software. Four random long-chained organisms per field were selected, and their lengths were measured along the long axis. The results revealed that the length of organisms in the Ha6, Ha5 and La4 groups was greater than that in the pH 7 group (*P* < 0.05, Fig. 2B).

#### Increased aggregation and hydrophobicity of planktonic *S. mutans* under early low pH shock

To evaluate changes in surface properties that promote biofilm formation, we measured bacterial aggregation and hydrophobicity. A significant increase in the aggregation rate of *S. mutans* was seen in several low pH groups after lactic and hydrochloric acid treatments, with significant differences in the Ha6, Ha4, and La5 groups. The aggregation rate of the hydrochloric acid Ha4 group was greater than that of Ha5 and Ha6, while the lactic acid La6 and La5 groups were greater than that of the La4 group (Fig. 2C). Notably, the La4 group, which exhibited the most pronounced growth impairment (Fig. 1A), also showed the lowest aggregation rate among the lactic acid-treated groups. The hydrophobicity of *S. mutans* was increased to varying degrees at early low pH, with the effect of hydrochloric acid being more pronounced (Fig. 2D).

### Biofilm biomass, morphology, protein content and EPS content of *S. mutans* under early low pH shock

**Fig. 3:**
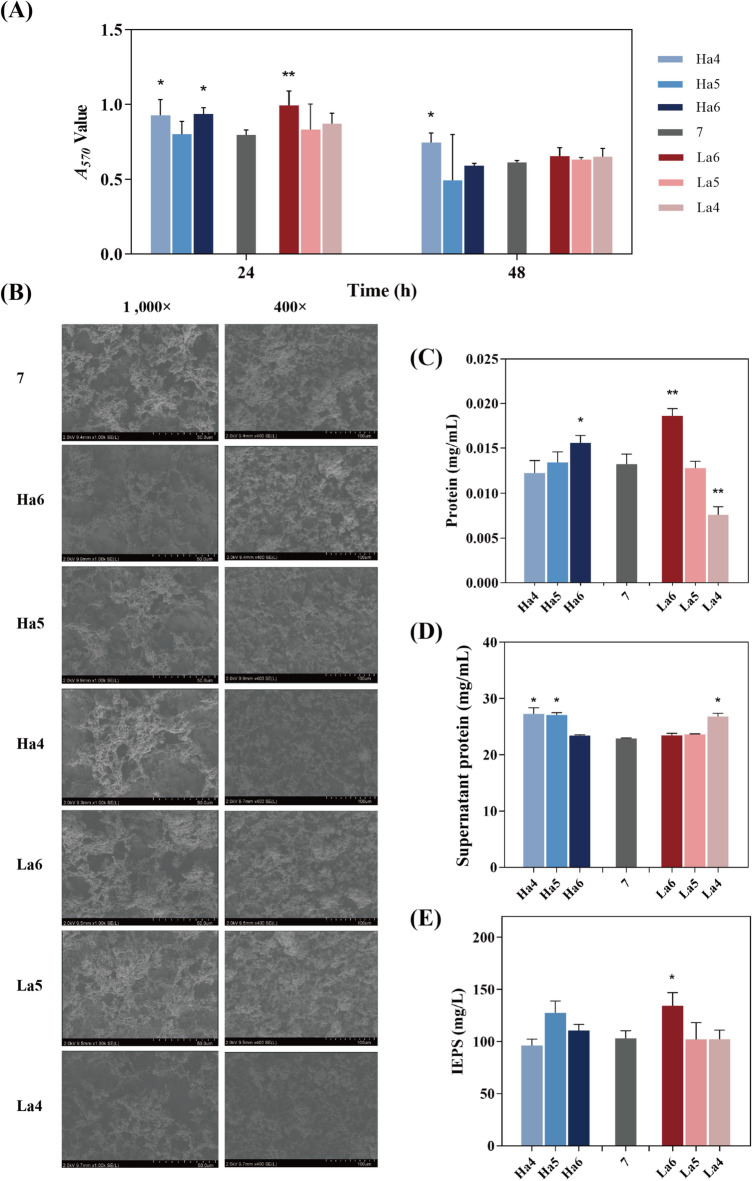
Biofilm biomass, morphology, EPS and protein contents of *S. mutans* under early low pH shock. (**A**) Crystalline violet staining to characterize 24 h and 48 h biofilm biomass; (**B**) Scanning electron microscopy at 1000× and 400× magnification to observe the biofilm morphology at 24 h; (**C**-**D**) Protein contents in *S. mutans* biofilms and supernatants under early low pH shock; (**E**) IEPS contents in *S. mutans* biofilms under early low pH shock. Comparison with the pH 7 group: *: *P* < 0.05, **: *P* < 0.01, ***: *P* < 0.001, not labelled i.e. no significant difference seen.

In order to further investigate the differences in the growth status of *S. mutans* under early low pH shock, we examined the biofilm biomass and observed the biofilm morphology. Crystalline violet staining showed Ha4/Ha6/La6 (24 h) and Ha4 (48 h) biofilm biomass greater than pH 7 group and others remained basically unchanged, which showed that the biofilm biomass did not fluctuate greatly under early low pH shock, and even some groups were slightly greater than the pH 7 group (Fig. 3A). Scanning electron microscope observation of biofilm morphology under 400x and 1000x microscope also revealed no significant differences among the groups (Fig. 3B). To further explore the biofilms, we also examined the protein content and EPS content in it. We found that the protein content in the biofilm under early low pH shock increased significantly at pH 6 (Ha6 and La6 groups), but decreased at pH 4 under lactic acid (La4 group), and the supernatant protein leakage increased (significant difference between Ha4, Ha5, and La4) (Fig. 3C, D). In addition, in all the lactate group biofilms, the insoluble exopolysaccharide (IEPS) increased significantly in the La6 group (Fig. 3E). In conclusion, no significant differences in the biofilm content or morphology of *S. mutans* were observed under early low pH shock, besides, the protein content and EPS content in the biofilm increased in some low pH groups.

#### PPI network analysis and pathway enrichment of DEGs

**Fig. 4:**
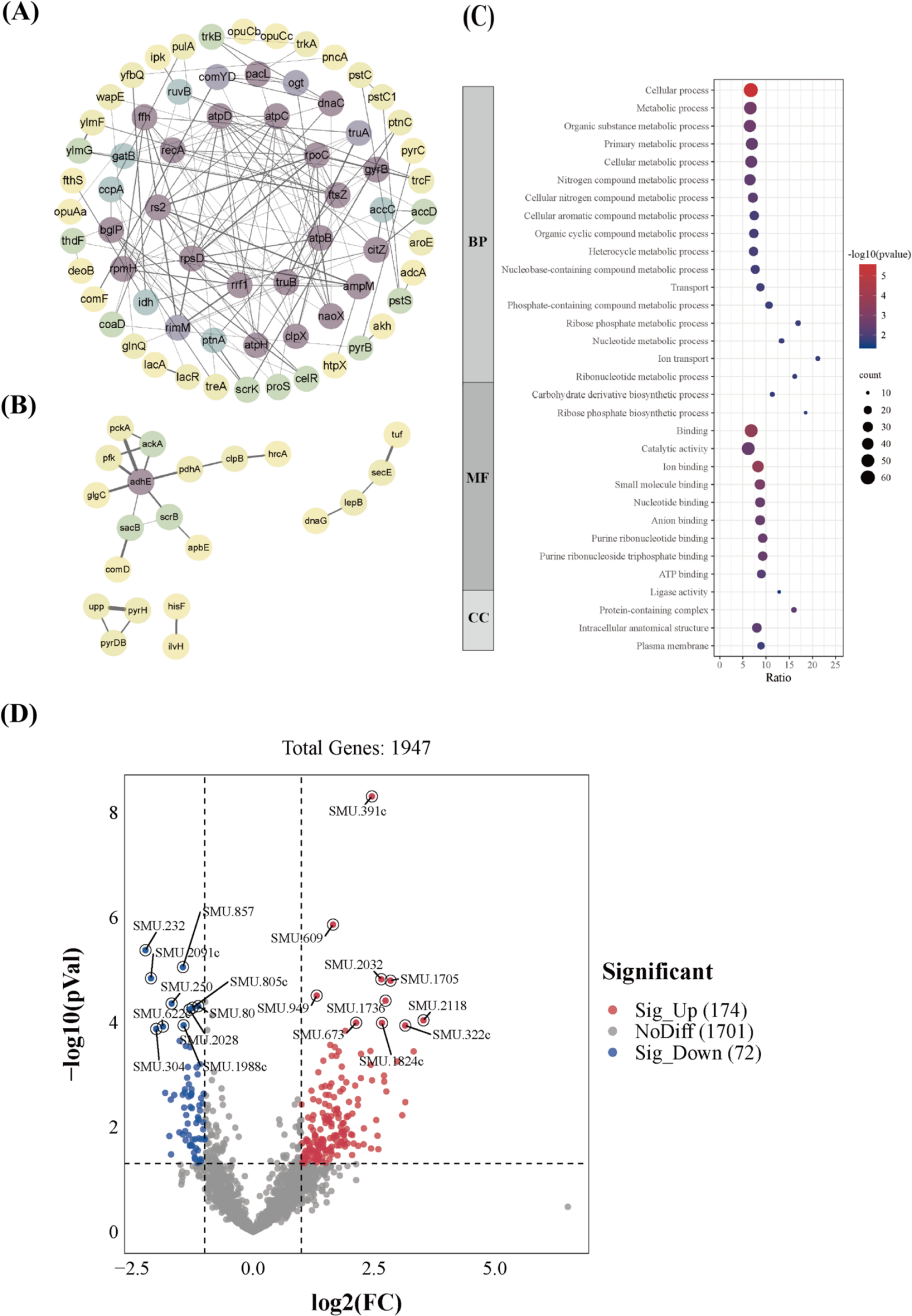
PPI network analysis and GO analysis based on DEGs. Each node represents a protein, and the color of the nodes is set according to the degree, the darker the color is, the greater the degree; the thickness of the edges is set according to the combined score, the thicker the line is, the greater the combined score. (**A**) Interaction network diagram of 76 proteins involved in protein interactions in proteins represented by significantly up-regulated genes; (**B**) Interaction network diagram of 21 proteins involved in protein interactions in proteins represented by significantly down-regulated genes. (**C**) Gene ontology categories of DEGs in molecular function (MF), cellular component (CC) and biology process (BP). (**D**) Volcano plot of gene regulation in *S. mutans* under different pH shock, blue represents down-regulated genes and red represents up-regulated genes. |Log2 (FC)| > 1, *P* < 0.05.

To identify key functional modules and interaction networks underlying the transcriptional response to acid shock, we analyzed differentially expressed genes (DEGs) in *S. mutans* at pH 5 compared to pH 7 using the GSE55952 dataset (|log2 FC| > 1, padjust < 0.05). Based on these DEGs, a protein-protein interaction (PPI) network was constructed using the STRING database, which included 76 proteins corresponding to up-regulated genes and 21 proteins corresponding to down-regulated genes (Fig. 4A, B). We listed the top ten nodes with the highest degree in Supplementary Tables 3, and the up-regulated proteins such as RpoC, Rs2, AtpD, and RpsD had the greatest degree, implying that they interacted with other proteins the most. Functional annotation of the DEGs was performed using GO enrichment analysis. GO enrichment analysis of molecular function (MF), biological process (BP) and cellular component (CC) indicated the involvement of these DEGs. Most DEGs were associated with ion transport and ribose phosphate metabolic processes in BP, carbohydrate derivative biosynthetic processes and ribose phosphate biosynthetic processes in MF, and protein-containing complexes in CC (Fig. 4C).

#### Differentially expressed genes of *S. mutans* in biofilms under early low pH shock

To characterize the global transcriptional response of *S. mutans* to early acid shock, we analyzed gene expression profiles at pH 5 compared to pH 7. The distribution of *S. mutans* gene expression between pH 5 stress and pH 7 is represented by a volcano plot (Fig. 4D). Compared with the pH 7 group, 72 significantly down-regulated genes (log2 (FC) < −1, padjust < 0.05) and 174 significantly up-regulated genes (log2 (FC) > 1, padjust < 0.05) were found in the pH 5 group. Among the top 10 up-regulated genes with the largest padjust and log2 (FC) were *opuCc*, *accC*, *rpsB*, *clpX*, etc., and the down-regulated genes were *hrcA*, *sacB*, *nifU* and *ilvH*, etc. (Supplementary Table 4), of which the complete list of genes for all up-regulated and down-regulated DEGs can be shown in Supplementary Tables 5 and 6.

#### RT-qPCR validation of several differentially expressed genes and other biofilm-related genes

**Fig. 5:**
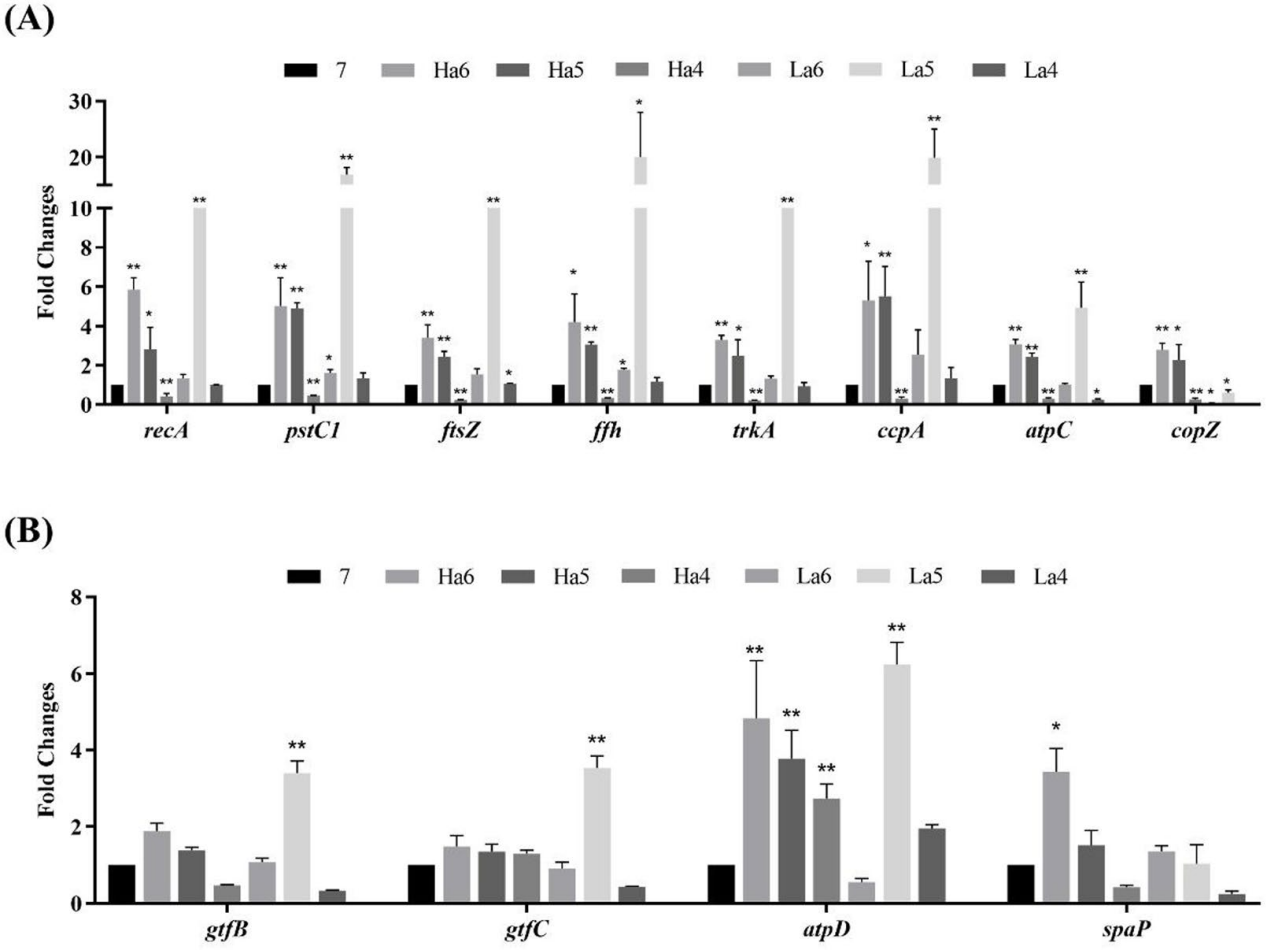
Validation of some differentially expressed genes and the expression of other biofilm-associated genes in 24 h *S. mutans* biofilm. (A) Validation of differentially expressed genes; (**B**) expression of biofilm-related genes. Comparison with the pH 7 group: *: *P* < 0.01, **: *P* < 0.001, not labelled i.e. no significant difference seen.

To validate the transcriptomic findings, we performed RT-qPCR on a panel of genes selected based on their significant differential expression and their documented roles in *S. mutans* virulence, including acid tolerance, biofilm formation, and cell morphology. Significant up-regulation of cariogenic genes (*recA*, *pstC1*, *ftsZ*, *ffh*, *trkA*, *ccpA*, *atpC*, *copZ*) and biofilm genes (*gtfB*, *gtfC*, *spaP*) was observed under low pH, peaking at pH 5 with lactic acid (Fig. 5A, B). Expression declined at pH 4, mirroring growth inhibition. In addition, the acid resistance-related gene *atpD* was significantly up-regulated in several low pH groups.

## Discussion

Dental caries imposes a major public-health burden^18^. The long - term impacts of pediatric caries can extend into adulthood, creating lasting health and economic risks^19^.The virulence of oral pathogenic flora may be influenced by their genetics, acid metabolism properties, and biofilm-forming ability, and morphological alterations may also play a role. Among oral microbial communities, *S. mutans* drives the disease through sucrose-dependent plaque formation and acid tolerance^20,21^. This prominence relies on the ability of *S. mutans* to survive in the acidic environments it generates, making its capacity to withstand acid stress crucial. This acidic stress closely mirrors the oral environment associated with pediatric caries, where children’s oral microflora is often disturbed by sugar-dense diets that stimulate bacterial acid production. Exploring the virulence and morphological changes of *S. mutans* under early low pH conditions not only contributes to our in-depth understanding of caries pathogenesis but also provides potential targets for developing customized preventive strategies for children who are particularly susceptible to dental caries.

Using lactic acid gradients we found growth inhibition only at pH 4, whereas pH 6–5 merely delayed log-phase entry; Hydrochloric acid showed no effect, highlighting lactic-acid-specific stress possibly linked to higher molar demand or yet-unknown factors. Aggregation and hydrophobicity govern short-range interactions driving bacterial adhesion and biofilm formation^22^. We found that early low-pH stress raised *S. mutans* aggregation and hydrophobicity, which may be due to more polysaccharides and proteins on the cell surface. The biofilm polysaccharide dextran, for example, induced aggregation of *Bacillus subtilis* (*B. subtilis*) and stimulated its adhesion to *S. mutans* biofilms^23^. In addition, we found that the *S. mutans* biofilm biomass did not decrease or even increased under early low pH shock, however, the contents of protein and extracellular polysaccharides within the biofilms were somewhat reduced by lactic acid but not by hydrochloric acid, which also corresponded to the growth curve results. Similarly, low pH environment was found to promote biofilm production in Group B *Streptococcus*, where the bacteria within the biofilm formed a high-density population that was more resistant to stress^24,25^. In order to understand this ‘paradoxical’ increase in biofilm biomass in *S. mutans* under early low pH shock, we analyzed the transcriptome of *S. mutans* after early low pH exposure using the GEO database. The results of the GO analysis revealed that ion transport and ribose phosphate metabolic process play important roles in low pH stress. In addition, acid tolerance-related genes, such as *atpD*, showed high connectivity in the PPI network, implying that they assume significant functions in bacterial resistance to low pH stress.

In our study, *recA* and *fstZ* were found to be significantly up-regulated in both the transcriptome and RT-qPCR validation. RecA assumes a crucial role in the bacterial stress response, where stagnant DNA replication forks and/or various forms of DNA damage under stress produce single-stranded DNA (ssDNA) fragments that activate the RecA recombinase into the RecA* state (Mg^2+^/ATP bound state) and consequently lead to increased GbpC production^26,27^. The increasing complexity of glucan-binding protein C (GbpC) lectin networks interacting with dextran polymers within biofilm matrices leads to structures that are difficult to disperse, as well as increased biofilm content and robustness^28,29^. Furthermore, it has been found that FtsZ depletion is lethal to *S. mutans*, leading to abnormal round cell shapes and microcell formation, suggesting an important role for FtsZ in maintaining cell shape stability^30^. It has also been reported that the polymerization of FtsZ in *S. mutans* is stronger in acidic environments than in neutral ones^31^. This finding was also consistent with the results of the present study.

Our study revealed a significant aspect ratio extension in *S. mutans* under early low pH shock in all groups except for the La4 group compared with that in a normal medium. It has been observed that *S. mutans* exhibits similar elongation patterns at different growth time scales, a phenomenon that has been suggested as a possible manifestation of multicellularity across various ecological niches^32^. It has also been shown that bacteria face DNA damage caused by antibiotic stress with a filamentous cellular morphology in which cell division is blocked, similar to the bacterial elongation, which improves bacterial survival under unfavorable conditions^33^. Bacterial elongation may confer selective advantages, including enhanced nutrient acquisition, improved dispersal, and reduced predation risk^32^. Such benefits also apply to bacteria that grow under specific or extreme conditions, where bacterial extensions can improve their ability to survive in harsh environments. This morphological change is particularly important in the oral environment. The elongation of oral bacteria may be a strategy for their adaptation to acidic environments that affects their formation and stability in biofilms.

One study revealed that bacteria with a low aspect ratio (spherical in shape) tend to form compact, spherical colonies when confined. Conversely, bacteria with a high aspect ratio (rod-shaped) push their offspring further out, resulting in elongated colonies that have a larger surface area. This, in turn, enhances their ability to acquire nutrients^34^. Similarly, we may be able to speculate that this bacterial elongation phenomenon present in *S. mutans* under early low pH shock is not just a stressful change, but may also play a contributory role for the increase in biofilms mentioned above. This is also consistent with the fact that the phenomenon was not observed in the La4 group and that the delayed entry into the logarithmic growth phase was associated with a decrease in the final bacterial concentration. It has also been shown that the aspect ratio of bacteria has a significant effect on their attachment behaviors in the flow. Bacteria with higher aspect ratios are more likely to be reorientated in the flow, thus attaching and colonizing the leeward side^35^.

Several limitations of this study should be acknowledged. First, as an in vitro study, our findings cannot fully replicate the complex oral environment, though this controlled approach was essential to establish causality. Second, the use of a single reference strain (UA159) may not capture strain-specific variations; future studies with clinical isolates are warranted. Third, our observation window focuses on early responses, leaving long-term adaptive mechanisms for future investigation. Finally, this study focused on *S. mutans* mono-species biofilms, which do not fully recapitulate the complex multi-species interactions present in the oral microbiome. Future investigations using mixed-species biofilm models are warranted to validate whether the observed morphological and transcriptional responses persist in a more clinically relevant context. Additionally, while we identified phenotypic and transcriptional changes, the precise molecular mechanisms linking acid shock to morphological regulation require further exploration, such as through gene knockout studies. These limitations notwithstanding, the present study provides a morphological and transcriptional foundation for understanding how early acid shock enhances the cariogenic potential of *S. mutans*.

The findings of this study offer several clinically relevant insights for pediatric dentistry. First, we provide a mechanistic basis for understanding why children with frequent dietary sugar exposure and consequently repeated acid shocks are at heightened caries risk, as early acid stress paradoxically primes *S. mutans* for enhanced biofilm formation through morphological adaptation. Additionally, our observation that virulence enhancement occurs during the delayed growth phase underscores the importance of early preventive interventions, such as biofilm disruption and dietary modification, before significant acid accumulation and bacterial proliferation occur. These translational aspects warrant further investigation in clinical settings and mixed-species biofilm models.

In summary, the present study established a gradient of early low pH shock to investigate the altered cariogenic potential in *S. mutans*. The findings indicate that early acid shock induces bacterial elongation in *S. mutans*, which may contribute to its cariogenic potential by enhancing biofilm formation and stability. These findings elucidate the morphological basis of how early acid shock fuels the cariogenic potential of *S. mutans* and provide a scientific rationale for developing antimicrobials that target the specific acidic and alkaline adaptations of this pathogen, potentially reducing the high incidence of dental caries in the pediatric population.

## Supplementary Information

Below is the link to the electronic supplementary material.

Supplementary Material 1

## Data Availability

The data supporting the results of the present study are available from the corresponding author upon reasonable request. All requests should be referred to the corresponding author for consideration.

## Declarations

### Supporting information

Additional supporting information can be found in the Supporting Information section. Supplementary Table 1 shows the pH changes in the pre-experiment. Supplementary Table 2 lists the gene primer sequence. Supplementary Table 3 presents the top 10 genes with the greatest degree in the PPI network of up and down. Supplementary Table 4 shows the details of the volcano plot of gene regulation in *S. mutans* under different pH stresses. And the complete list of all up- and down-regulated DEGs can be found in Supplementary Tables 5 and 6.
