# Supplementary material for: Acid shock triggers virulence surge accompanied by bacterial elongation in streptococcus mutans
Source: Sci Rep. 2026 May 12;16:21677. doi: 10.1038/s41598-026-52966-x (PMC13357595; doi:10.1038/s41598-026-52966-x)
Supplement: Supplementary file 1 — Supplementary Material 1 [file 41598_2026_52966_MOESM1_ESM.pdf]

Supplementary Table 1. pH changes in the pre-experiment

Time (h)	pH
0 h	7.32
0 h	7.14
0 h	7.27
3 h	6.57
3 h	6.39
3 h	6.52
6 h	5.32
6 h	5.34
6 h	5.67
9 h	4.73
9 h	4.63
9 h	4.78
12 h	4.25
12 h	4.32
12 h	4.17
24 h	4.08
24 h	4.02
24 h	4.11
48 h	4.07
48 h	4.01
48 h	4.03

Supplementary Table 2. Gene Primer Sequence

**Gene name**	**Primer sequence (5′-3′)**	
	**Forward**	**Reverse**
16s rRNA	AGCGTTGTCCGGATTTATTG	CTACGCATTTCACCGCTACA
atpD	TGTTGATGGTCTGGGTGAAA	TTTGACGGTCTCCGATAACC
gtfB	CACTATCGGCGGTTACGAAT	CAATTTGGAGCAAGTCAGCA
gtfC	GATGCTGCAAACTTCGAACA	TATTGACGCTGCGTTTCTTG
sapP	TCCGCTTATACAGGTCAAGTTG	GAGAAGCTACTGATAGAAGGGC
copZ	TATCATATTGATGGCTTAAAATGC	TTTCCTGTAATCCTGACTTCTTTT
ffh	AAATGATTCCTGGTATGGC	CTCGGAGTTAGGAGGTCAG
pstC1	TTGTGGTTCCGTTTGTGAG	GCAAGACTGGCTTCCTTAT
atpC	GGGTTGCAGTTAATGGTGG	CGTTCTGCCCGAGAAAGAT
ftsZ	CGACCTGATTACAAGTCCA	ACCAGTACCGATACCGATT
ccpA	TAAGAAAGTCCCAGAAGAC	CATAGCAACTGCTCCTAAA
trkA	GGTCGTATTGAAACTTTGC	ATCATCACCAGTAGGGAAA
recA	TAAGATTGCCAGTGATTTG	ATTGAACACGCACTTTATG

Supplementary Table 3. The top 10 genes with the greatest degree in the PPI network of up and down.

	Gene ID	node	node_degree
**Up (top 10)**			
	SMU.1989	*rpoC*	16
	SMU.2032	*rs2*	16
	SMU.1530	*atpD*	15
	SMU.2135c	*rpsD*	14
	SMU.1624	*rrf1*	12
	SMU.1528	*atpB*	11
	SMU.1529	*atpC*	10
	SMU.552	*ftsZ*	10
	SMU.2085	*recA*	10
	SMU.1144	*truB*	10
**Down (top 10)**			
	SMU.148	*adhE*	7
	SMU.1978	*ackA*	3
	SMU.2028	*sacB*	3
	SMU.1843	*scrB*	3
	SMU.1425	*clpB*	2
	SMU.1188	*lepB*	2
	SMU.1389	*pckA*	2
	SMU.1423	*pdhA*	2
	SMU.1191	*pfk*	2
	SMU.1223	*pyrDB*	2

Supplementary Table 4. Details of volcano plot of gene regulations in *S. mutans* under different pH stresses.

	Gene ID	Gene name	Gene description	Log2FC	-Log10P
**Up Gene**					
	SMU.2118	*opuCc*	putative ABC transporter	3.5201898	4.0361849
	SMU.322c	*NA*	glucose-1-phosphate uridylyltransferase	3.1438923	3.9372056
	SMU.1560	*NA*	hypothetical protein	3.0827039	2.2254525
	SMU.1705	*NA*	hypothetical protein	2.8412402	4.7935263
	SMU.1736	*accC*	acetyl-CoA carboxylase biotin carboxylase subunit	2.7370037	4.4111396
	SMU.1824c	*NA*	transcriptional repressor CodY	2.6636334	3.9872207
	SMU.2032	*rpsB*	30S ribosomal protein S2	2.6502408	4.8200741
	SMU.673	*NA*	hypothetical protein	2.1297189	3.9905696
	SMU.609	*NA*	putative 40K cell wall protein precursor	1.6523093	5.8629771
	SMU.391c	*NA*	hypothetical protein	2.4557308	8.3083737
	SMU.949	*clpX*	ATP-dependent protease ATP-binding subunit ClpX	1.3144847	4.5081323
**Down Gene**					
	SMU.80	*hrcA*	heat-inducible transcription repressor	-1.134816	4.3071813
	SMU.805c	*NA*	putative amino acid ABC transporter	-1.25225	4.2756439
	SMU.2028	*sacB*	levansucrase precursor	-1.315882	4.2397035
	SMU.1988c	*NA*	putative DNA binding protein	-1.437111	3.9410076
	SMU.857	*NA*	putative uracil permease	-1.45052	5.0514649
	SMU.250	*nifU*	putative nitrogen fixation-like protein, NifU	-1.684595	4.3569444
	SMU.622c	*NA*	hypothetical protein	-1.863019	3.9174742
	SMU.304	*NA*	putative deaminase	-2.002824	3.8724966
	SMU.2091c	*NA*	DNA mismatch repair protein MutS	-2.111444	4.8389611
	SMU.232	*ilvH*	acetolactate synthase 3 regulatory subunit	-2.224954	5.3739306

Supplementary Table 5. 174 up DEGs

Gene ID	FC	p	symbol	Common Name of Primary Target
SMU.2118	11.4731512	9.20E-05	*opuCc*	putative ABC transporter, osmoprotectant-binding protein, glycine betaine/carnitine/choline ABC transporter
SMU.151	9.971683641	0.000360227		hypothetical protein
SMU.427	8.854506038	0.003348509	*copZ*	putative copper chaperone
SMU.322c	8.839055972	0.000115557		glucose-1-phosphate uridylyltransferase
SMU.1560	8.472007581	0.005950418		hypothetical protein
SMU.1060	7.888604725	0.000561242	*ffh*	signal recognition particle protein subunit, Ffh
SMU.1705	7.16635834	1.61E-05		hypothetical protein
SMU.996	6.749898202	0.002095995		ferrichrome ABC transporter permease protein
SMU.1736	6.666842687	3.88E-05	*accC*	acetyl-CoA carboxylase biotin carboxylase subunit
SMU.941c	6.540333425	0.001352782		hypothetical protein
SMU.1912c	6.524815354	0.001043594		hypothetical protein
SMU.1824c	6.336268198	0.000102986		transcriptional repressor CodY
SMU.2032	6.277720682	1.51E-05	*rpsB*	30S ribosomal protein S2
SMU.1783	6.015492912	0.026528846	*proS*	prolyl-tRNA synthetase
SMU.1137	5.948542643	0.014654728	*pstC1*	putative phosphate ABC transporter, permease protein
SMU.1529	5.838365483	0.00735687	*atpC*	F0F1 ATP synthase subunit gamma
SMU.770c	5.495991236	0.00128295		putative manganese transporter
SMU.391c	5.485909254	4.92E-09		hypothetical protein
SMU.552	5.420625335	0.025647485	*ftsZ*	cell division protein FtsZ
SMU.1735	5.403558111	0.000647631	*accD*	acetyl-CoA carboxylase subunit beta

SMU.1519	5.371766459	0.000355024	*glnQ*	putative amino acid ABC transporter, ATP-binding protein
SMU.168	4.887662251	0.010602698		putative transcriptional regulator
SMU.1144	4.873846222	0.021385365	*truB*	tRNA pseudouridine synthase B
SMU.1591	4.694133629	0.000401413	*ccpA*	catabolite control protein A, CcpA
SMU.1706	4.684685503	0.00116442		hypothetical protein
SMU.227c	4.508644321	0.022887795		hypothetical protein
SMU.167	4.496864922	0.002939456		hypothetical protein
SMU.776	4.480478847	0.003839097		hypothetical protein
SMU.673	4.376322035	0.000102195		hypothetical protein
SMU.1950	4.316676703	0.001618044		putative pseudouridylate synthase
SMU.229	4.228682813	0.020645149		hypothetical protein
SMU.1619c	4.147832985	0.005537354		putative metalloprotease
SMU.1840	4.145813241	0.014203869	*scrK*	putative fructokinase
SMU.2037	4.132542698	0.034167528	*treA*	putative trehalose-6-phosphate hydrolase TreA
SMU.1909c	4.06620095	0.013714843		hypothetical protein
SMU.1722c	3.952840024	0.00331547		putative integral membrane protein
SMU.940c	3.905978166	0.00613846		putative hemolysin III
SMU.1117	3.868246599	0.020540879	*naoX*	NADH oxidase (H2O-forming)
SMU.866	3.821757136	0.010924061		hypothetical protein
SMU.1483c	3.819243132	0.006449322		hypothetical protein
SMU.1214	3.802373899	0.001401462	*pyrC*	dihydroorotase
SMU.2117	3.752655985	0.000146107	*opuCb*	putative osmoprotectant ABC transporter, permease protein

SMU.1358	3.734141708	0.023440857		putative putative transposase
SMU.1652	3.719674117	0.018076321	*ogt*	putative methylated-DNA--protein-cysteine S-methyltransferase
SMU.1091	3.69429022	0.012012754	*wapE*	cell wall protein, WapE
SMU.423	3.682916119	0.014183809		hypothetical protein
SMU.781	3.681816133	0.02113772		prephenate dehydrogenase
SMU.541	3.664115651	0.014241703		hypothetical protein
SMU.2026c	3.655966647	0.008351915		30S ribosomal protein S10
SMU.1798c	3.617343547	0.001886514		hypothetical protein
SMU.1708	3.594091543	0.008496542	*trkA*	potassium transporter peripheral membrane component
SMU.1745c	3.569946987	0.012377625		putative transcriptional regulator
SMU.340	3.561035109	0.000702413	*rpmH*	50S ribosomal protein L34
SMU.1063	3.550960507	0.001143335	*opuAa*	putative ABC transporter, ATP-binding protein, proline/glycine betaine transport system
SMU.1100c	3.550667347	0.012329353		putative permease
SMU.2109	3.54307395	0.019480503		putative MDR permease, multidrug efflux pump
SMU.1646c	3.531113647	0.018684313		hemolysis inducing protein
SMU.925	3.50350555	0.009867908		hypothetical protein
SMU.1561	3.48783116	0.002098025	*trkB*	putative potassium uptake system protein TrkB
SMU.1983	3.444612057	0.022522819	*comYD*	putative competence protein ComYD
SMU.1163c	3.413365662	0.005550864		putative ABC transporter, ATP-binding protein
SMU.1073	3.413056328	0.000360307	*fthS*	formate--tetrahydrofolate ligase
SMU.872	3.40081779	0.006691918		putative PTS system, fructose-specific enzyme IIABC component

SMU.498	3.354144003	0.034724569	*comF*	putative late competence protein
SMU.671	3.348804253	0.014732619	*citZ*	citrate synthase
SMU.853	3.337688571	0.004450626	*lspA*	putative lipoprotein signal peptidase
SMU.1496	3.325794858	0.022336151	*lacA*	galactose-6-phosphate isomerase subunit LacA
SMU.228	3.325692227	0.004765164		putative alkaline-shock protein
SMU.2079c	3.299079203	0.00043847		hypothetical protein
SMU.1527	3.280890392	0.014278963	*atpC*	F0F1 ATP synthase subunit epsilon
SMU.87	3.27551126	0.005538763		hypothetical protein
SMU.995	3.259573318	0.017518423		ferrichrome ABC transporter permease protein
SMU.554	3.225815491	0.000297725	*ylmF*	hypothetical protein
SMU.1022	3.201619965	0.004667129	*citX*	2'-(5''-triphosphoribosyl)-3'-dephospho-CoA:apo- citrate lyase
SMU.1432c	3.193323899	0.014434467		putative endoglucanase precursor
SMU.980	3.191913257	0.002691269	*bglP*	putative PTS system, beta-glucoside-specific EII component
SMU.1877	3.160649622	0.016003713	*ptnA*	putative PTS system, mannose-specific component IIAB
SMU.609	3.143363811	1.37E-06		putative 40K cell wall protein precursor
SMU.803c	3.093628673	0.032856227		putative ABC transporter, ATP-binding protein
SMU.1871c	3.088531561	0.000438588		hypothetical protein
SMU.639	3.084511148	0.023644922		putative acetyltransferase
SMU.1724c	3.075918687	0.008539076		putative rRNA methylase
SMU.1521	3.071391139	0.009698492		putative amino acid ABC transporter, permease protein
SMU.67	3.067921437	0.001471485		putative acyltransferase
SMU.501	3.062003312	0.028846597		hypothetical protein

SMU.150	3.058470768	0.006243718		hypothetical protein
SMU.728	3.02263425	0.025707137		putative oxidoreductase
SMU.2085	3.022060586	0.000271642	*recA*	recombinase A
SMU.162c	3.007183584	0.016312353		hypothetical protein
SMU.1823	2.996060868	0.001697308	*pncA*	putative pyrazinamidase/nicotinamidase
SMU.1541	2.990883378	0.04098182	*pulA*	putative pullulanase
SMU.1802c	2.977898852	0.007835504		hypothetical protein
SMU.1230c	2.971901676	0.00395998		hypothetical protein
SMU.1748	2.963402731	0.002447503	*akh*	aspartate kinase
SMU.1323	2.959797215	0.00705902		hydrolase
SMU.517	2.949176658	0.007387283	*coaD*	phosphopantetheine adenylyltransferase
SMU.1235	2.897742078	0.006154884	*trmE*	tRNA modification GTPase TrmE
SMU.125	2.871724831	0.027689223		hypothetical protein
SMU.1624	2.836883863	0.031659871	*frr*	ribosome recycling factor
SMU.1908c	2.832260498	0.002418583		hypothetical protein
SMU.145	2.825713395	0.048054182		hypothetical protein
SMU.1599	2.820489333	0.008358239	*celR*	putative transcriptional regulator, possible antiterminator
SMU.672	2.811136598	0.001641864	*idh*	isocitrate dehydrogenase
SMU.974	2.81039438	0.007260938	*potB*	putative spermidine/putrescine ABC transporter, permease protein
SMU.1574c	2.804974037	0.003614556		hypothetical protein
SMU.1996	2.794913325	0.027225032	*ipk*	4-diphosphocytidyl-2-C-methyl-D-erythritol kinase
SMU.385	2.78306737	0.006597465		putative glycoprotein endopeptidase

SMU.555	2.743009743	0.018184712	*ylmG*	hypothetical protein
SMU.1797c	2.735896368	0.000541851		hypothetical protein
SMU.1826	2.735436777	0.021355432	*yfbQ*	aminotransferase AlaT
SMU.86	2.722431656	0.024365673		hypothetical protein
SMU.1587c	2.719244163	0.017104408		hypothetical protein
SMU.1479	2.706809671	0.00539607		hypothetical protein
SMU.1767c	2.697593066	0.038939056		hypothetical protein
SMU.1277	2.679993949	0.046046463	*gyrB*	DNA gyrase subunit B
SMU.523	2.676375983	0.001729373		hypothetical protein
SMU.1498	2.66750802	0.018082698	*lacR*	lactose repressor
SMU.1429	2.641198355	0.017123343	*murC2*	putative UDP-N-acetylmuramyl tripeptide synthetase MurC
SMU.281	2.62365271	0.020080659		hypothetical protein
SMU.750c	2.620331535	0.011087859		hypothetical protein
SMU.1556	2.598866701	0.024961479	*ampM*	methionine aminopeptidase
SMU.991	2.586067212	0.03407571		putative ribonucleotide reductase
SMU.2138	2.564673417	0.003198906	*dnaC*	replicative DNA helicase
SMU.1704	2.557133023	0.021948273		hypothetical protein
SMU.84	2.556158793	0.036346455	*truA*	tRNA pseudouridine synthase A
SMU.1563	2.54557647	0.000900995	*pacL*	putative cation-transporting P-type ATPase PacL
SMU.632	2.543327635	0.038791658		putative transcriptional regulator
SMU.797	2.54078427	0.027856267		hypothetical protein
SMU.1989	2.540157776	0.042040602	*rpoC*	DNA-directed RNA polymerase subunit beta'

SMU.1302	2.517854055	0.01934315	*adcA*	putative surface adhesin, AdcA protein homolog, putative Zn-binding lipoprotein
SMU.1040c	2.517662672	0.02205844		putative oxidoreductase, short-chain dehydrogenase/reductase
SMU.331	2.50560096	0.033435172		putative transcriptional regulator
SMU.1534	2.498722926	0.036722193	*atpH*	F0F1 ATP synthase subunit C
SMU.92c	2.488991243	0.047525742		putative putative transposase
SMU.949	2.487134799	3.10E-05	*clpX*	ATP-dependent protease ATP-binding subunit ClpX
SMU.399	2.473669032	0.029921569		hypothetical protein
SMU.1800c	2.440986467	0.014569472		hypothetical protein
SMU.890	2.435235656	0.040113548		hypothetical protein
SMU.1781	2.42951536	0.044212595		hypothetical protein
SMU.1929	2.426930059	0.015779722	*htpX*	heat shock protein HtpX
SMU.1260c	2.424781033	0.014127784		hypothetical protein
SMU.2096c	2.392758445	0.028848479		hypothetical protein
SMU.1819	2.3852889	0.019594099	*gatB*	aspartyl/glutamyl-tRNA amidotransferase subunit B
SMU.1138	2.372660177	0.005956616	*pstS*	putative ABC transporter, phosphate-binding protein
SMU.318	2.367888302	0.038878853		putative hippurate hydrolase
SMU.942	2.367815064	0.011535867	*mvaA*	putative hydroxymethylglutaryl-CoA reductase
SMU.1878	2.366921973	0.002949943	*ptnC*	putative PTS system, mannose-specific component IIC
SMU.08	2.343466185	0.007206199	*trcF*	putative transcription-repair coupling factor
SMU.1973	2.331899869	0.027145154	*pepA*	putative glutamyl-aminopeptidase, endo-1,4-beta-glucanase
SMU.124	2.329777249	0.030983012		MarR family transcriptional regulator
SMU.1914c	2.306576375	0.002025521		hypothetical protein

SMU.64	2.29251355	0.010015809	*ruvB*	Holliday junction DNA helicase RuvB
SMU.867	2.262817089	0.02438642	*rimM*	16S rRNA-processing protein RimM
SMU.858	2.240216925	0.030615678	*pyrB*	aspartate carbamoyltransferase catalytic subunit
SMU.1504c	2.23717932	0.021975124		hypothetical protein
SMU.262	2.220962496	0.042564484	*otcA*	putrescine carbamoyltransferase
SMU.1528	2.215910297	0.027475725	*atpB*	F0F1 ATP synthase subunit beta
SMU.1292c	2.201906109	0.006579612		hypothetical protein
SMU.1809	2.199435758	0.018378957	*scnG*	putative bacteroiocin operon protein ScnG-like protein
SMU.1233	2.167427163	0.037293786	*deoB*	phosphopentomutase
SMU.915c	2.16638135	0.037064231		7-cyano-7-deazaguanine reductase
SMU.239c	2.157045563	0.018282299		hypothetical protein
SMU.778	2.151410473	0.018494537	*aroE*	shikimate 5-dehydrogenase
SMU.841	2.143670687	0.017138805		putative aminotransferase
SMU.1020	2.122933463	0.028419809	*cilB*	putative citrate lyase CilB, citryl-CoA lyase, beta subunit
SMU.1253c	2.109226642	0.040308055		hypothetical protein
SMU.2135c	2.098621605	0.030954766	*rpsD*	30S ribosomal protein S4
SMU.1530	2.087781413	0.048934777	*atpD*	F0F1 ATP synthase subunit alpha
SMU.811	2.0644142	0.045476387		hypothetical protein
SMU.926	2.057198232	0.038724885		GTP-pyrophosphokinase
SMU.1136	2.036875348	0.03602672	*pstC*	putative phosphate ABC transporter, permease protein
SMU.60	2.027660201	0.020156443		DNA alkylation repair enzyme
SMU.1109c	2.022291133	0.047823163		putative integral membrane protein, permease

SMU.2077c	2.001941858	0.003714187	hypothetical protein

Supplementary Table 6. 72 down DEGs

Gene ID	FC	p	symbol	Common Name of Primary Target
SMU.232	0.213905547	4.22736E-06	*ilvH*	acetolactate synthase 3 regulatory subunit
SMU.2091c	0.231415312	1.4489E-05		DNA mismatch repair protein MutS
SMU.304	0.249511064	0.000134123		putative deaminase
SMU.622c	0.274900433	0.000120928		hypothetical protein
SMU.2046c	0.2838516	0.002230668		hypothetical protein
SMU.1072c	0.301341488	0.014870732		putative acetyltransferase
SMU.1659c	0.30766832	0.00300211		hypothetical protein
SMU.1423	0.30889193	0.033248866	*pdhA*	putative pyruvate dehydrogenase, TPP-dependent E1 component alpha-subunit
SMU.250	0.311090136	4.39598E-05	*nifU*	putative nitrogen fixation-like protein, NifU
SMU.1397c	0.322803749	0.002449616		hypothetical protein
SMU.821	0.347931184	0.012640711	*dnaG*	DNA primase
SMU.913	0.349093299	0.000226996		glutamate dehydrogenase
SMU.714	0.364892172	0.013829112	*tuf*	elongation factor Tu
SMU.863	0.365040832	0.002411537		putative ABC transporter, ATP-binding protein
SMU.857	0.36588952	8.8825E-06		putative uracil permease
SMU.1988c	0.369306186	0.000114549		putative DNA binding protein
SMU.2119	0.372252209	0.004256735	*opuCd*	putative osmoprotectant ABC transporter, permease protein
SMU.1183	0.375774016	0.002195636	*mtlA2*	PTS system, mannitol-specific enzyme IIA
SMU.532	0.376340985	0.00191229	*trpE*	anthranilate synthase component I
SMU.392c	0.377979914	0.011947056		hypothetical protein
SMU.816	0.380440598	0.017864818		transaminase

SMU.148	0.381554643	0.000283929	*adhE*	bifunctional acetaldehyde-CoA/alcohol dehydrogenase
SMU.669c	0.383660706	0.005905751		putative glutaredoxin
SMU.1088	0.388452758	0.001145094	*apbE*	putative thiamine biosynthesis lipoprotein
SMU.10	0.391792471	0.008590482		hypothetical protein
SMU.1538	0.39291278	0.000716181	*glgC*	glucose-1-phosphate adenylyltransferase
SMU.179	0.396966293	0.00262739		hypothetical protein
SMU.394c	0.397560627	0.014232408		hypothetical protein
SMU.363	0.398309109	0.001526219	*glnR*	transcriptional regulator, glutamine synthetase repressor
SMU.2028	0.401679883	5.75833E-05	*sacB*	levansucrase precursor, beta-D-fructosyltransferase
SMU.905	0.404064743	0.022690797		putative ABC transporter, ATP-binding protein
SMU.1902c	0.406033464	0.02200483		hypothetical protein
SMU.1625	0.406295082	0.000300201	*pyrH*	uridylate kinase
SMU.614	0.407490558	0.004135297		hypothetical protein
SMU.1916	0.409925715	0.00240222	*comD*	putative histidine kinase of the competence regulon, ComD
SMU.1344c	0.410065989	0.0025076		putative malonyl-CoA acyl-carrier-protein transacylase
SMU.191c	0.410493554	0.022031931		putative integrase
SMU.747c	0.41426504	0.001903289		putative permease
SMU.1946	0.414446349	0.002286011		hypothetical protein
SMU.2090c	0.416233366	0.004367681		hypothetical protein
SMU.1656	0.418868022	0.022665627	*serC*	phosphoserine aminotransferase
SMU.1622	0.419297039	0.017404942	*pmsR*	methionine sulfoxide reductase A
SMU.805c	0.419792855	5.30098E-05		putative amino acid ABC transporter, ATP-binding protein

SMU.1188	0.429421417	0.030813418	*lepB*	putative signal peptidase
SMU.1502c	0.433419479	0.038267194		hypothetical protein
SMU.1670c	0.434290557	0.040688468		hypothetical protein
SMU.1191	0.435219794	0.017075253	*pfkA*	6-phosphofructokinase
SMU.1310	0.441637982	0.025381791		hypothetical protein
SMU.1948	0.44313491	0.008414758	*secE*	preprotein translocase subunit SecE
SMU.252	0.444999092	0.000726163		hypothetical protein
SMU.1195	0.451683429	0.04804872		permease
SMU.1843	0.451991853	0.000988937	*scrB*	sucrose-6-phosphate hydrolase
SMU.217c	0.452992248	0.027782805		hypothetical protein
SMU.1241	0.454086179	0.006456749	*uvrC*	excinuclease ABC subunit C
SMU.80	0.455393063	4.92968E-05	*hrcA*	heat-inducible transcription repressor
SMU.1142c	0.456798348	0.007367049	*spxA*	transcriptional regulator Spx
SMU.1978	0.457517442	0.006944463	*ackA*	putative acetate kinase
SMU.368c	0.459000554	0.016525162		hypothetical protein
SMU.1961c	0.461674713	0.003879453		putative PTS system, sugar-specific enzyme IIA component
SMU.1223	0.466813523	0.004817124	*pyrDB*	dihydroorotate dehydrogenase 1B
SMU.1673	0.467189721	0.000634483	*upp*	uracil phosphoribosyltransferase
SMU.1264	0.469319616	0.04339714	*hisF*	imidazole glycerol phosphate synthase subunit HisF
SMU.963c	0.471504772	0.0408886		putative deacetylase
SMU.1059	0.473029385	0.045211087	*satC*	hypothetical protein
SMU.412c	0.473672311	0.002811915		putative Hit-like protein involved in cell-cycle regulation

SMU.1200	0.475700252	0.017006788	*rpsA*	30S ribosomal protein S1
SMU.143c	0.485849759	0.003527265	*def*	peptide deformylase
SMU.308	0.488869634	0.011049991		sorbitol-6-phosphate 2-dehydrogenase
SMU.1389	0.489122083	0.008137563	*pckA*	hypothetical protein
SMU.889	0.492769551	0.002303936	*pbpX*	putative penicillin-binding protein, class C, fmt-like protein
SMU.1425	0.499751625	0.016735318	*clpB*	putative Clp proteinase, ATP-binding subunit ClpB
SMU.767	0.499778723	0.017161104		putative transposase, ISSmu1
